# The Dark Triad of Personality’s Relationship with Compliance towards COVID-19 Pandemic Recommendations along with Anxiety and Depressive Symptoms in Polish Citizens

**DOI:** 10.3390/ijerph18105478

**Published:** 2021-05-20

**Authors:** Anna M. Gogola, Paweł Dębski, Agnieszka Goryczka, Piotr Gorczyca, Magdalena Piegza

**Affiliations:** Chair and Clinical Department of Psychiatry, Faculty of Medical Sciences in Zabrze, Medical University of Silesia, 42-612 Tarnowskie Góry, Poland; pdebski@sum.edu.pl (P.D.); agnieszka.goryczka14@gmail.com (A.G.); pgorczyca@sum.edu.pl (P.G.); mpiegza@sum.edu.pl (M.P.)

**Keywords:** Dark Triad, COVID-19 restrictions, pandemic, HADS, depression, anxiety

## Abstract

The outbreak of the COVID-19 pandemic forced everyone to comply with rules of a sanitary regime and social distancing on a daily basis. The aim of our research was to assess the differences in the levels of Dark Triad traits between people who obeyed and disobeyed the pandemic restrictions. Additionally, we considered the possible correlation between the Dark Triad and the intensity of symptoms of depression and anxiety. A total of 604 Polish participants, whose average age was 28.95 ± 11.27 years, completed an online survey which measured Dark Triad traits using the Polish version of the Dirty Dozen test. Anxiety and depression were assessed using the Hospital Anxiety and Depression Scale (HADS). The results revealed a possible relationship between personality traits and compliance with pandemic restrictions. Individuals with higher levels of psychopathy tended to disobey newly introduced rules. On the other hand, a higher level of subclinical narcissism might have contributed to a better civil compliance. The results showed a significant positive correlation between the intensity of the Dark Triad and the occurrence of depressive symptoms. Furthermore, narcissism was linked to anxiety symptoms. These results can contribute to a better understanding of behavioural patterns during the COVID-19 pandemic within the group of individuals who exhibit the Dark Triad traits. Our conclusions might help to identify individuals who are particularly vulnerable to mental health problems.

## 1. Introduction

The outbreak of the COVID-19 pandemic in 2020 forced many countries all over the world to combat the fast-spreading virus. The Polish authorities decided to introduce pandemic restrictions on an unprecedented scale (isolation, quarantine, social distancing, and the shutdown of many economic sectors). The priority was to reduce the spread of SARS-CoV-2 caused by people with symptoms of COVID-19 as well as by asymptomatic or pre-symptomatic individuals [1].

Anderson et al. claimed that the compliance of individuals with COVID-19 rules was crucial to control the course of the coronavirus pandemic [2]. This theory was considered most pertinent to western European countries. Citizens of those countries are used to many civil rights and liberties which are an essential part of their everyday existence. Many of them exhibit hostile attitudes towards pandemic restrictions. They took matters into their own hands to safeguard their civil liberties by disobeying the pandemic rules imposed by EU governments. This resistance could be observed in many countries all over the world and it took various forms. The characteristics of people who disobeyed pandemic restrictions have been a subject of many studies [3,4,5,6,7,8,9,10]. It is very important to examine and understand motives underlying this disobedience due to the fact that results of many studies have shown that the risk of infection sinks when individuals comply with pandemic recommendations [11,12]. The factors which can predict obedience or disobedience are belief in the effectiveness of pandemic recommendations, trust in governments’ decisions [3], individuals’ particular ways of perceiving a given situation [4,5], and their personality [3,4,5,6,7,8,9,10]. Great trust in the government and seeing oneself as more vulnerable to COVID-19 infection [3] or perceiving the situation as dangerous [5], negative, or as demanding duty [4] will encourage compliance towards recommendations. The characteristics of one’s personality type are related to the way one perceives the outer world [13], which means that different individuals can have different views regarding the same situation. Individuals with low levels of certain Big Five personality traits (conscientiousness or agreeableness) [4,5,6,7] as well as individuals with high levels of Dark Triad personality traits [4,8,9,10] tended to experience more difficulties obeying pandemic recommendations. People with a significant intensity of Dark Triad traits are known to experience pleasure at another’s misfortune and exhibit a tendency to engage in controversial and antisocial behaviours [14]. Therefore, it is very unlikely that such individuals would decide to modify their everyday behaviour to protect the health of others during the COVID-19 pandemic. Furthermore, even if such individuals decide to react to the new situation, they are more likely to focus on behaviours like hoarding and less on preventive precautions [8].

The construct known as the Dark Triad of Personality [15] is widely used in studies which explore negative and undesirable behaviours [8,9,10,14,16,17]. Due to the common core of subclinical psychopathy, subclinical narcissism, and Machiavellianism, it is possible to combine them together in the analysis of socially aversive personalities. [18] All components can be characterized by egoism and egocentrism, a tendency to exhibit antisocial and unethical behaviour [16,17], limited empathy [19], low agreeableness [15], and a difficulty in understanding and expressing emotions (alexithymia) [20]. Psychopathy can be characterised as a tendency to oppose the rest of society and to exhibit impulsive and reckless behaviours [21]. Both psychopathy and Machiavellianism have a strong correlation with low conscientiousness [15]. Individuals who exhibit a higher intensity of this trait tend to use manipulative techniques to their personal gain at the expense of others [22]. Narcissism can be considered as overexpression of one’s uniqueness which causes a strong need of social exposure [23]. Furthermore, narcissistic individuals struggle to accept criticism, experience feelings of self-doubt, and usually are focused on building a positive social image of themselves. Narcissism can take different forms (e.g., grandiose and vulnerable, agentic and communal) [23]. Moreover, it is considered the least destructive of all Dark Triad traits [24] and can encourage prosocial behaviours [25].

The SARS-CoV-2 virus can pose a serious threat to mental health. This threat can come not only from the infection itself (e.g., phobias, eating disorders) [26] but also from further-reaching consequences of the pandemic restrictions [27] and the way in which they have impacted our everyday lives. Some of the pandemic recommendations (e.g., stay at home recommendations, avoidance to social gathering and cultural activities, social distancing) are linked to many stress factors (e.g., domestic violence, worsening domestic finances) [27] and correlate with a wide variety of negative emotions including elevated stress levels, anxiety, and depression [28]. Soon after the outbreak of COVID-19, Galea [29] claimed that it is necessary to introduce measures aimed at the prevention of mental health problems during the pandemic. Previous measures such as telepsychology, improving access to information, and financial support [27] seem to be insufficient because the prevalence of mental disorders keep rising [30]. Special care should be provided for particularly vulnerable groups (medical care workers, children, students, psychiatric patients) [27]. It is important to remember that one’s personality can also impact this vulnerability [31]. A high level of Machiavellianism correlates positively with depressive and anxiety symptoms [24,32,33,34,35]. Although psychopathy is associated with low anxiety [36], a study by Derefinko [37] claims that the relationship between anxiety and psychopathy is more complex and that linking psychopathy to a lack of anxiety is not correct [38]. Many studies have shown that psychopathy correlates positively with anxiety and depression [24,32,35]. Narcissism is linked to certain positive attributes which can prevent individuals from excessive stress, depression, or anxiety [39,40] and therefore contribute to maintaining good mental health. However, the relationship between narcissism and depression is more complicated and depends on the type of narcissism [32,41,42], with some types (e.g., grandiose) being prone to negative emotions [42,43]. This study is an attempt to consider whether individuals who exhibit a high intensity of the Dark Triad traits might be one of the groups particularly vulnerable to mental health problems triggered by the pandemic. We still lack studies which could confirm or deny this hypothesis. Our current study is an attempt to fill in this gap.

The aim of this study was to assess the differences in the Dark Triad of personality between people who obeyed and disobeyed pandemic restrictions and to analyse the relationship of its component traits with the severity of depressive and anxiety symptoms. In our current research, we tried to consider the threat which people with Dark Triad traits could pose on public health and security. But furthermore, we wanted to indicate that people with a high intensity of Dark Triad traits could be in danger of suffering from negative mental health consequences themselves. This approach lets us adapt a more holistic view on the Dark Triad of personality.

## 2. Materials and Methods

### 2.1. Data and Participants

The Dark Triad of personality, anxiety, and depression were measured using the convenience sampling method (sampling via social media). For this purpose, an online survey was created, consisting of a sociodemographic questionnaire and psychological scales—the Dirty Dozen and the Hospital Anxiety and Depression Scale (HADS). The survey was created using Google Forms software and shared via social media networks (Facebook and Instagram) from the 10th of November until 2 December 2020. On 27 October 2020, Polish authorities introduced lockdown type measures for the second time in Poland. They lasted for the whole of November and December. During this time, citizens were obliged to stay at least 1.5 m away from other people and to wear a face mask as well as to follow hand hygiene recommendations and restrict their movement. A total of 604 Polish citizens participated in the study—468 women (77.50%) and 136 men (22.50%), aged 28.95 ± 11.27 years. Respondents did not receive any award for their participation in our research. Our survey did not allow participants to provide incomplete responses. Moreover, the answers provided by each one of the respondents were analysed separately, and their accuracy was checked by the authors of this study. Due to this, all responses were valid and could be included in the study. Table 1 presents sociodemographic characteristics of the study group.

### 2.2. Measures

#### 2.2.1. The Dirty Dozen

Jonason and Webster’s questionnaire [44] is well-known and widely used as a scientific research tool for assessing the intensity of the Dark Triad of personality. It consists of 12 questions. The intensity of each trait (psychopathy, narcissism, and Machiavellianism) is assessed using 4 questions, and the respondents provide their answers on a 5-point scale (from 1 to 5). The results can be considered either as a whole or divided into subscales of psychopathy, narcissism, and Machiavellianism. The minimum score possible to obtain in each subscale is 4 (12 for whole), while the maximum is 20 (60 for whole). The Polish version of the questionnaire was created in 2016 by Czarna [45] by adapting Jonason and Webster’s original version. The validation analysis confirmed that the measure met the criteria of psychometric usefulness with good reliability ranging between 0.67 and 0.79 [45], making it a reliable research tool. In our study, the Cronbach’s alpha coefficient for the whole test was estimated at 0.85.

#### 2.2.2. The Hospital Anxiety and Depression Scale (HADS)

The scale by Zigmond and Snaith [46] is commonly used to assess anxiety and depressive symptoms for healthy individuals as well as for people who suffer from somatic disorders. The test consists of a total of 14 questions which are divided into two subscales: the anxiety subscale (HADS-A) and the depression subscale (HADS-D). Each subscale contains 7 questions which assess respondents’ moods over the past week. The answers are given on a 4-point scale (from 0 to 3). The scores obtained on each subscale are summed (the minimum score possible to each subscale is 0, while the maximum is 21). A meta-analysis by Bjelland et al. [47] has shown that scores ≥ 8 are most often “an optimal cut-off score for both HADS-A and HADS-D”. Adhering to this conclusion, we defined all scores ≥ 8 on the HADS-A or HADS-D scales as significant levels of depressive or anxiety symptoms. The anxiety and depressive symptom severity scores are analysed as follows [48]: 0–7 points—no anxiety or depression, 8–10—mild anxiety or depression, 11–14—moderate anxiety or depression, 15–21—severe anxiety or depression. The high sensitivity and specificity of the subscales (about 0.80) [47] make the scale a reliable screening tool. In our study, the Cronbach’s alpha coefficient for the whole test was 0.80.

#### 2.2.3. Adherence to the Recommendations

In order to divide our respondents into two groups, we asked them 3 yes-or-no questions: “Do you follow hand hygiene recommendations?”; “Do you follow social distancing recommendations?”; “Did you go out less often during the COVID-19 pandemic?” The responses to each of the three questions were analysed separately, and we performed a triple division of our respondents. Participants who answered “yes” were considered as those adhering to one of the three recommendations, and those who answered “no” were considered as those who did not adhere.

### 2.3. Data Analysis

The analysis of the collected data was performed using the computer programs Excel 2016 and Statistica version 13.3. For the measures used, Cronbach’s alpha coefficients in the study sample were determined. The normality of the distributions was assessed based on the Shapiro–Wilk test. We calculated the mean levels of Dark Triad considered both as a whole and in its components, as well as levels of depressive and anxiety symptoms among those who adhered to a specific recommendation and those who did not. We compared the results from both groups for each of the three recommendations. Due to the size of the groups being compared (each group > 100), the *t*-test was used [49] to test the significance of intergroup differences, assuming the statistical significance level *p* ≤ 0.05. We analysed the correlations between the Dark Triad of Personality, its components and the symptoms of anxiety and depression in the respondents. Correlation matrices were prepared using Spearman’s rank correlation coefficient, where *p* < 0.05 was taken as the level of statistical significance. To complete the results, we added an assessment of the severity of anxiety and depressive symptoms among our participants.

### 2.4. Ethics

The study was approved by the university Bioethics Committee, and it adhered to the Declaration of Helsinki. Each participant was informed about the anonymity and voluntariness of participation in the study and had the opportunity to discontinue participation in the study at any time.

## 3. Results

Table 2 presents descriptive statistics of the variables. We included both women and men in the statistical analyses. For control purposes, a study of the significance of differences between gender groups was performed. The differences turned out to be typical. Men presented a significantly higher intensity of the overall Dark Triad score (28.235 vs. 24.620; *p* < 0.000) and its components—psychopathy (9.507 vs. 7.765; *p* < 0.000) and Machiavellianism (8.485 vs. 7.186; *p* < 0.000), while women presented a higher intensity of anxiety (9.278 vs. 7.794; *p* < 0.000).

The respondents were divided into groups according to whether they adhered to the recommendation to stay at home or not (Table 3). Those adhering to the recommendation to reduce the frequency of leaving their homes exhibited different intensity of psychopathy and narcissism compared to those not adhering to this recommendation. A higher level of psychopathy was exhibited by the group opposed to this recommendation. Interestingly, a higher level of narcissism was found in the group of respondents declaring a reduction in the frequency of leaving home.

Participants were also divided into groups based on whether or not they complied with hand hygiene recommendations (Table 4). Individuals who did not follow the recommendation to frequently disinfect their hands had a significantly higher level of psychopathy. On the other hand, those who adhered to this recommendation presented higher intensity of narcissism.

Lastly, the respondents were divided into those who adhered to social distancing restriction and those who did not (Table 5). Again, those declaring adherence to such restriction exhibited significantly different levels of psychopathy to those declaring non-adherence to the restriction. The opponents of social distancing had a higher average intensity of psychopathy.

We also took into consideration the relationships between the components of the Dark Triad of personality and the severity of the symptoms of anxiety and depression in the studied group (Table 6). The study revealed a significant positive correlation between the intensity of the Dark Triad, its components, and the severity of depressive symptoms. Narcissism also correlated positively with anxiety. Significant correlations were also observed between the Dark Triad components: at a moderate level between psychopathy and Machiavellianism and narcissism and at a high level between narcissism and Machiavellianism. Finally, the severity of anxiety symptoms in the study group exhibited a moderate, positive correlation to the severity of depressive symptoms.

**Figure 1:**
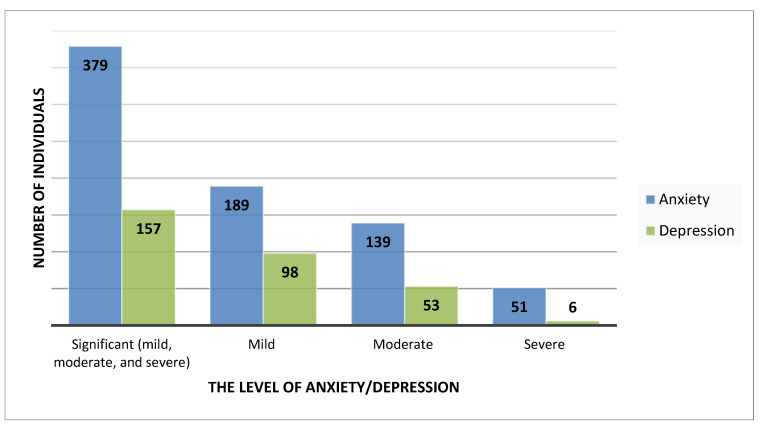
The level of anxiety and depressive symptoms in the entire study group (*n* = 604).

Interesting results were also obtained in terms of depressive and anxiety symptoms in the entire study group (Figure 1). The mean severity of anxiety among the study group was 8.929 ± 3.710 points. Significant levels of anxiety symptoms were observed in 379 individuals (mild—189, moderate—139, severe—51) which equals 26% of the study group. In the study group, the mean severity of depressive symptoms was 5.512 ± 3.388 points. Significant levels of depressive symptoms were observed in 157 individuals (mild—98, moderate—53, severe—6) which equals 63% of the study group.

## 4. Discussion

In our study, we examined differences in the Dark Triad traits among two groups of respondents—individuals who adhered to recommendations and individuals who did not. In order to draw reliable conclusions about the relationship between Dark Triad and behavioural tendencies, it is necessary to consider each trait of this cluster individually. The intensities of subclinical narcissism and psychopathy seem to have exhibited opposite effects on the behaviours of individuals during the pandemic. Narcissism can encourage compliance towards pandemic restrictions (e.g., hand hygiene recommendations, social distancing). On the other hand, individuals with higher levels of psychopathy are more likely to disobey those recommendations. The results obtained in our study did not show any significant correlation between Machiavellianism and civil compliance. Nonetheless, other authors claimed that this kind of connection indeed exists and that all of the three Dark Triad traits are linked to disobedience towards pandemic restrictions [4,8,9].

Bearing in mind that each one of the Dark Triad traits is actually a complex set of various characteristics in itself, the specific similarities and differences between psychopathy and narcissism must be considered. Narcissism as well as psychopathy is linked to extraversion and openness [15]. Previous studies showed that individuals with higher extraversion tended to have difficulties with obeying social distancing rules. On the other hand, this trait did not impact compliance with the hand hygiene recommendations [7]. The observations made by Zajenkowski et al. [4] showed a negative correlation between extraversion and compliance towards pandemic restrictions. These observations contradict the conclusions drawn by Aschwanden et al. [6]—here higher extraversion was associated with higher obedience. Despite common features shared by narcissism and psychopathy, the key role in their impact on one’s behaviour is in all probability played by the specific interactions between the different traits which, as a whole, form the psychopathic and narcissistic tendency.

The behaviour of individuals who score higher on narcissism is based on external sources of motivation and their actions are usually aimed at building a positive social image of themselves. This makes them tend towards prosocial behaviours [25]. It is very possible that the adoption by narcissistic individuals of a certain behavioural pattern is caused by the intensified need of building and maintaining a positive social image (e.g., being an upstanding citizen who obeys pandemic restrictions). Vulnerable narcissism is linked to a significant tendency to feel shame [23] and therefore an inability to take criticism from other members of society (e.g., being criticised due to disobedience towards pandemic restrictions).

In contrast to narcissism, psychopathy is linked to low conscientiousness [15], which can also affect one’s ability to follow the new everyday duties of the strict pandemic rules. Furthermore, low neuroticism can cause diminished ability to experience emotions like anxiety or shame [36,50] and is specific to individuals who exhibit a higher level of subclinical psychopathy [15]. Therefore, low neuroticism can predispose these individuals to ignore the necessity of following safety measures during the pandemic [5]. Psychopathic individuals are also more immune to criticism (because of their low ability to feel guilt or shame [50]) and can therefore easily break social norms by disobeying newly introduced rules. The diminished ability to fear the possible consequences of getting a SARS-CoV-2 infection can also intensify the tendency to disobey.

Another difference between psychopathy and narcissism is the tendency to exhibit antisocial behaviours [14,16,17]. Positive correlation with this trait is significantly stronger in psychopathy than in narcissism [16]. Some authors claim that narcissism is not at all connected with a tendency to exhibit socially undesirable behaviours [51]. The particular characteristics of psychopathic personalities which are linked to antisocial behaviours, immorality, and a lack of empathy, can additionally encourage disobedience towards pandemic restrictions.

Our study showed that the impact which the Dark Triad might have on civil compliance cannot be considered as purely negative. Its impact seems to be dualistic: narcissism can be a trait which encourages compliance while psychopathy can be linked-to disobedience.

In the current study, we also decided to examine the relationship between the Dark Triad and anxiety and depression within society during the pandemic. Depressive symptoms were present in 26% of respondents among the study group, and 63% experienced a significant severity of anxiety symptoms during the pandemic. These scores might have been influenced by the negative impact of stress factors linked to the COVID-19 pandemic. Before the pandemic, the prevalence of mental disorders in which anxiety and depressive symptoms were present in Polish society was determined as follows: major depression—3%, minor depression—0.4%, and anxiety disorders such as panic attacks—6.2%, generalised anxiety disorder—1.1%, social phobias—1.4%, specific phobias—3.4% [52]. However, among students (who made up about 45% of our respondents), the prevalence of depression was higher [53]. Due to the limitations of our study (selection of a sample, the HADS, which cannot be used for epidemiological studies), our results are not representative of the general population and should be interpreted in the context of the characteristics of our study group. Due to the high number of scientific studies which examine mental health during the pandemic, we decided to focus on the relationship between the Dark Triad traits and depressive and anxiety symptoms.

According to our best knowledge, this study is the first to focus on the relationship between the Dark Triad traits and symptoms of anxiety and depression during the pandemic. The results obtained showed that the Dark Triad as well as all of its individual components correlated positively with depressive symptoms. Mood disorders are a result of emotional dysregulation [54], which is commonly exhibited by narcissistic, psychopathic, and Machiavellian individuals [55,56]. Additionally, psychopathy and Machiavellianism correlate with a reluctance to seek social support even during difficult, stressful life situations [57]. Attempting to endure life’s difficulties without seeking from other members of society can have a negative impact on mental well-being [58]. Psychopathy and Machiavellianism are linked to a deficiency in task-oriented coping skills, especially in terms of dealing with stressful situations [57]. Difficulties in solving problems may lead to negative mental health consequences. Machiavellianism correlates positively with anhedonia (which can one of the symptoms of depression) and alexithymia [33], and this relationship might explain the occurrence of mood disorders. Although previous studies claimed that narcissism can prevent from depression [39] our study showed a positive correlation between depressive symptoms and narcissism. Narcissism is associated with emotional instability [55] and correlates negatively with mental toughness [42]. Furthermore, vulnerable narcissism is bound up with negative emotions (such as stress [42]) and decreased mood [55]. Therefore, this type of narcissism is more strongly linked to depressive disorders [43]. We think it might be true that among our study group this type of narcissistic tendency was particularly common. We would like to suggest that future studies on the correlation between mental well-being and the Dark Triad traits should use methods which distinguish between different types of narcissism.

In our study, subclinical narcissism was positively correlated with anxiety symptoms. Psychopathy and Machiavellianism were not shown to have had any statistically significant correlations with levels of anxiety. During the COVID-19 pandemic individuals with higher levels of narcissism have been observed to develop a negative affect [10] which underlies emotional disorders such as anxiety [54]. With regard to narcissism, the vulnerable type seems in this case to be the most relevant. It can be characterised by an inability to cope with failure and criticism, low self-esteem [23], and reduced psychological resilience [42], which can intensify the feeling of insecurity and lead to emotional dysregulation [55]. Additionally, vulnerable narcissism is associated with high neuroticism [59]. Individuals who exhibit these traits will be more prone to experience emotional tension [60]. Neurotic individuals may tend to follow the news about the development of the pandemic very carefully and get strongly engaged in searching for the latest information and updates [61], which may cause a significant increase in perceived anxiety and depression [62]. This behaviour can pose a serious threat to the mental well-being of these individuals.

Some previous studies also showed the negative impact of the Dark Triad traits on mental health [24,32,35], and our research confirmed these results. However, the relationship between the Dark Triad of personality and mental health is not fully resolved issue. Different authors claim that a high level of narcissism may also have some positive influence [39,40] on the mental health of individuals, which was not confirmed by our study. Therefore, we believe that this issue still needs further research in order to draw reliable conclusions.

## 5. Limitations

The present study has some limitations which future research could aim at improving. Firstly, the method of convenience sampling we used is flawed, and it does not ensure a random selection of a sample. The study was carried out on social media networks. Therefore, our sample consists only of individuals who have internet access and an account on those social media networks. For these reasons the results of our study may not be relevant for the whole population. Furthermore, convenience sampling does not provide total control over the age and gender of respondents. The cause of this limitation is that within our respondents were 28 people aged 16 and 17. Due to the fact that this age is not an obstacle in measuring levels of personality traits, we decided to keep their answers in our study. Secondly, the Dirty Dozen test does not distinguish between different subtypes of narcissism, psychopathy, and Machiavellianism. Thirdly, the questions used to assess civil compliance with pandemic restrictions were originally established by the authors of this study. The answers given were based on self-report methods and they should not be considered as an objective evaluation of the participants’ actions. Fourthly, in our analysis, we considered a whole study group without dividing it according to age and gender. The fact that there was a big difference between the number of male and female participants, as well as that young adults predominated, might have biased the results. Therefore, we would like to suggest that the results of our study are most relevant for young, well-educated adults, especially women. Future studies should consider the age, gender, and education diversity of the study group.

## 6. Conclusions

1. Individuals with a high intensity of psychopathy may exhibit reluctance to comply with the rules of the sanitary regime.
2. Subclinical narcissism may be linked to a need of adjustment to newly introduced rules.
3. Individuals with high intensity of Dark Triad traits may require special psychological and psychiatric care during this health crisis because they might be more vulnerable to depressive symptoms.
4. Subclinical narcissism correlates positively with anxiety symptoms.

## Figures and Tables

**Table 1:** Sociodemographic characteristics of the study group.

	Number of Individuals	% of Total Number
**Study group**	604	100%
**Gender:**		
• **Female**	468	77.50%
• **Male**	136	22.50%
**Age (years):**		
• 16–19	60	10.00%
• 20–29	360	60.00%
• 30–39	51	8.00%
• 40–49	87	14.50%
• 50+	46	7.50%
**Place of residence:**		
• Village	193	32.00%
• City with <50,000 inhabitants	128	21.00%
• City with 50,000–100,000 inhabitants	62	10.00%
• City with >100,000 inhabitants	221	37.00%
**Education:**		
• Currently studying	288	48.00%
• Tertiary	217	36.00%
• Secondary	85	14.00%
• Primary and vocational	14	2.00%

**Table 2:** Descriptive statistics of the variables.

	Mean	SD	Median	CI−95%	CI+95%
Dark Triad	25.434	8.290	24.000	7.847	8.786
Psychopathy	8.157	3.089	8.000	2.924	3.274
Narcissism	9.798	3.913	10.000	3.704	4.147
Machiavellianism	7.479	3.401	7.000	3.219	3.604
Anxiety	8.944	3.695	9.000	3.498	3.916
Depression	5.512	3.388	5.000	3.207	3.591

Notes: SD—standard deviation; CI—confidence interval.

**Table 3:** Differences between people declaring adherence to recommendations related to restricting leaving home and those who do not.

	Adhering to Restrictions (n = 439)		Non-Adhering to Restrictions (n = 165)		*t* Test	
	**Mean**	**SD**	**Mean**	**SD**	***t***	***p***
**Dark Triad**	25.453	7.990	25.382	9.065	0.094	0.925
**Psychopathy**	7.993	2.992	8.594	3.304	−2.136	0.033 *
**Narcissism**	10.000	3.800	9.261	4.163	2.075	0.038 *
**Machiavellianism**	7.460	3.327	7.527	3.598	−0.216	0.829
**Anxiety**	9.082	3.686	8.576	3.706	1.502	0.134
**Depression**	5.497	3.328	5.552	3.553	−0.177	0.859

Notes: values are significant at * *p* ≤ 0.05; SD—standard deviation.

**Table 4:** Differences between those who reported adherence to hand disinfection recommendations as often as possible and those who did not.

	Adhering to Recommendation (n = 465)		Non-Adhering to Recommendation (n = 139)		*t* Test	
	Mean	SD	Mean	SD	*t*	*p*
**Dark Triad**	25.424	7.881	25.468	9.563	−0.055	0.956
**Psychopathy**	7.983	2.955	8.741	3.448	−2.551	0.011 *
**Narcissism**	9.968	3.757	9.230	4.361	1.954	0.050 *
**Machiavellianism**	7.473	3.357	7.496	3.554	−0.071	0.943
**Anxiety**	9.026	3.605	8.669	3.984	0.999	0.318
**Depression**	5.492	3.341	5.576	3.555	−0.253	0.800

Notes: values are significant at * *p* ≤ 0.05; SD—standard deviation.

**Table 5:** Differences between individuals declaring adherence and non-adherence to social distancing restriction.

	Adhering to Distance (n = 442)		Non-Adhering to Distance (n = 162)		*t* Test	
	Mean	SD	Mean	SD	*t*	*p*
**Dark Triad**	25.362	8.130	25.630	8.735	−0.351	0.726
**Psychopathy**	7.989	3.047	8.617	3.165	−2.222	0.027 *
**Narcissism**	9.966	3.710	9.340	4.401	1.746	0.081
**Machiavellianism**	7.407	3.389	7.673	3.435	−0.850	0.396
**Anxiety**	9.020	3.688	8.735	3.720	0.842	0.400
**Depression**	5.380	3.395	5.870	3.355	−1.577	0.115

Notes: values are significant at * *p* ≤ 0.05; SD—standard deviation.

**Table 6:** Associations between the Dark Triad of personality, its components, symptoms of anxiety and depression in the entire study group.

	Dark Triad	Psychopathy	Narcissism	Machiavellianism	Anxiety	Depression
**Dark Triad**	1.000	0.683 **	0.825 **	0.823 **	0.064	0.160 **
**Psychopathy**		1.000	0.291 **	0.424 **	−0.063	0.10 6*
**Narcissism**			1.000	0.559 **	0.123 *	0.118 *
**Machiavellianism**				1.000	0.073	0.155 **
**Anxiety**					1.000	0.444 **
**Depression**						1.000

Notes: values are significant at * *p* < 0.05; at ** *p* < 0.001.

## Data Availability

Data supporting reported results are available on request from the corresponding author.

## References

[1] Wiersinga W.J., Rhodes A., Cheng A.C., Peacock S.J., Prescott H.C. (2020). Pathophysiology, Transmission, Diagnosis, and Treatment of Coronavirus Disease 2019 (COVID-19). JAMA.

[2] Anderson R.M., Heesterbeek H., Klinkenberg D., Hollingsworth T.D. (2020). How will country-based mitigation measures influence the course of the COVID-19 epidemic?. Lancet.

[3] Clark C., Davila A., Regis M., Kraus S. (2020). Predictors of COVID-19 voluntary compliance behaviors: An international investigation. Glob. Transit..

[4] Zajenkowski M., Jonason P.K., Leniarska M., Kozakiewicz Z. (2020). Who complies with the restrictions to reduce the spread of COVID-19?: Personality and perceptions of the COVID-19 situation. Personal. Individ. Differ..

[5] Abdelrahman M. (2020). Personality Traits, Risk Perception, and Protective Behaviors of Arab Residents of Qatar During the COVID-19 Pandemic. Int. J. Ment. Health Addict..

[6] Aschwanden D., Strickhouser J.E., Sesker A.A., Lee J.H., Luchetti M., Stephan Y., Sutin A.R., Terracciano A. (2020). Psychological and Behavioural Responses to Coronavirus Disease 2019: The Role of Personality. Eur. J. Personal..

[7] De Carvalho L.F., Pianowski G., Gonçalves A.P. (2020). Personality differences and COVID-19: Are extroversion and conscientiousness personality traits associated with engagement with containment measures?. Trends Psychiatry Psychother..

[8] Nowak B., Brzóska P., Piotrowski J., Sedikides C., Żemojtel-Piotrowska M., Jonason P.K. (2020). Adaptive and maladaptive behavior during the COVID-19 pandemic: The roles of Dark Triad traits, collective narcissism, and health beliefs. Personal. Individ. Differ..

[9] Triberti S., Durosini I., Pravettoni G. (2021). Social distancing is the right thing to do: Dark Triad behavioral correlates in the COVID-19 quarantine. Personal. Individ. Differ..

[10] Hardin B.S., Smith C.V., Jordan L.N. (2021). Is the COVID-19 pandemic even darker for some? Examining dark personality and affective, cognitive, and behavioral responses to the COVID-19 pandemic. Personal. Individ. Differ..

[11] Chu D.K., Akl E.A., Duda S., Solo K., Yaacoub S., Schünemann H.J. (2020). Physical distancing, face masks, and eye protection to prevent person-to-person transmission of SARS-CoV-2 and COVID-19: A systematic review and meta-analysis. Lancet.

[12] Hillier M.D. (2020). Using effective hand hygiene practice to prevent and control infection. Nurs. Stand..

[13] Jonason P., Sherman R. (2020). Personality and the perception of situations: The Big Five and Dark Triad traits. Personal. Individ. Differ..

[14] James S., Kavanagh P.S., Jonason P.K., Chonody J.M., Scrutton H.E. (2014). The Dark Triad, schadenfreude, and sensational interests: Dark personalities, dark emotions, and dark behaviors. Personal. Individ. Differ..

[15] Paulhus D.L., Williams K.M. (2002). The Dark Triad of personality: Narcissism, Machiavellianism, and psychopathy. J. Res. Pers..

[16] Moor L., Anderson J.R. (2019). A systematic literature review of the relationship between dark personality traits and antisocial online behaviours. Personal. Individ. Differ..

[17] Roeser K., McGregor V.E., Stegmaier S., Mathew J., Kübler A., Meule A. (2016). The Dark Triad of personality and unethical behavior at different times of day. Personal. Individ. Differ..

[18] Bertl B., Pietschnig J., Tran U.S., Stieger S., Voracek M. (2017). More or less than the sum of its parts? Mapping the Dark Triad of personality onto a single Dark Core. Personal. Individ. Differ..

[19] Jonason P.K., Lyons M., Bethell E.J., Ross R. (2013). Different routes to limited empathy in the sexes: Examining the links between the Dark Triad and empathy. Personal. Individ. Differ..

[20] Jonason P.K., Krause L. (2013). The emotional deficits associated with the Dark Triad traits: Cognitive empathy, affective empathy, and alexithymia. Personal. Individ. Differ..

[21] Fox S., Hammond S. (2017). Investigating the multivariate relationship between impulsivity and psychopathy using canonical correlation analysis. Personal. Individ. Differ..

[22] Abell L., Brewer G., Qualter P., Austin E. (2016). Machiavellianism, emotional manipulation, and friendship functions in women’s friendships. Personal. Individ. Differ..

[23] Sedikides C. (2021). In Search of Narcissus. Trends Cogn. Sci..

[24] Jonason P.K., Baughman H.M., Carter G.L., Parker P. (2015). Dorian Gray without his portrait: Psychological, social, and physical health costs associated with the Dark Triad. Personal. Individ. Differ..

[25] Kauten R.L., Barry C.T. (2016). Adolescent narcissism and its association with different indices of prosocial behavior. J. Res. Personal..

[26] AlSamman M., Caggiula A., Ganguli S., Misak M., Pourmand A. (2020). Non-respiratory presentations of COVID-19, a clinical review. Am. J. Emerg. Med..

[27] Pedrosa A.L., Bitencourt L., Fróes A.C.F., Cazumbá M.L.B., Campos R.G.B., de Brito S.B.C.S., Simões e Silva A.C. (2020). Emotional, Behavioral, and Psychological Impact of the COVID-19 Pandemic. Front. Psychol..

[28] Salari N., Hosseinian-Far A., Jalali R., Vaisi-Raygani A., Rasoulpoor S., Mohammadi M., Rasoulpoor S., Khaledi-Paveh B. (2020). Prevalence of stress, anxiety, depression among the general population during the COVID-19 pandemic: A systematic review and meta-analysis. Glob. Health.

[29] Galea S., Merchant R.M., Lurie N. (2020). The Mental Health Consequences of COVID-19 and Physical Distancing: The Need for Prevention and Early Intervention. JAMA Intern. Med..

[30] Généreux M., Schluter P.J., Landaverde E., Hung K.K., Wong C.S., Mok C.P.Y., Blouin-Genest G., O’Sullivan T., David M.D., Carignan M.-E. (2021). The Evolution in Anxiety and Depression with the Progression of the Pandemic in Adult Populations from Eight Countries and Four Continents. Int. J. Environ. Res. Public Health.

[31] Boyce P., Parker G., Barnett B., Cooney M., Smith F. (1991). Personality as a Vulnerability Factor to Depression. Br. J. Psychiatry.

[32] Gómez-Leal R., Megías-Robles A., Gutiérrez-Cobo M.J., Cabello R., Fernández-Abascal E.G., Fernández-Berrocal P. (2019). Relationship between the Dark Triad and depressive symptoms. PeerJ.

[33] Al Aïn S., Carré A., Fantini-Hauwel C., Baudouin J.-Y., Besche-Richard C. (2013). What is the emotional core of the multidimensional Machiavellian personality trait?. Front. Psychol..

[34] Bianchi R., Mirkovic D. (2020). Is Machiavellianism associated with depression? A cluster-analytic study. Personal. Individ. Differ..

[35] Sabouri S., Gerber M., Lemola S., Becker S.P., Shamsi M., Shakouri Z., Bahmani D.S., Kalak N., Holsboer-Trachsler E., Brand S. (2016). Examining Dark Triad traits in relation to sleep disturbances, anxiety sensitivity and intolerance of uncertainty in young adults. Compr. Psychiatry.

[36] Dolan M.C., Rennie C.E. (2007). Is juvenile psychopathy associated with low anxiety and fear in conduct-disordered male offenders?. J. Anxiety Disord..

[37] Derefinko K.J. (2015). Psychopathy and Low Anxiety: Meta-Analytic Evidence for the Absence of Inhibition, Not Affect. J. Personal..

[38] Visser B.A., Ashton M.C., Pozzebon J.A. (2012). Is Low Anxiety Part of the Psychopathy Construct?. J. Personal..

[39] Lyons M., Evans K., Helle S. (2019). Do “Dark” Personality Features Buffer Against Adversity? The Associations Between Cumulative Life Stress, the Dark Triad, and Mental Distress. SAGE Open.

[40] Akehurst S., Thatcher J. (2010). Narcissism, social anxiety and self-presentation in exercise. Personal. Individ. Differ..

[41] Fang Y., Niu Y., Dong Y. (2021). Exploring the relationship between narcissism and depression: The mediating roles of perceived social support and life satisfaction. Personal. Individ. Differ..

[42] Papageorgiou K.A., Gianniou F.-M., Wilson P., Moneta G.B., Bilello D., Clough P.J. (2019). The bright side of dark: Exploring the positive effect of narcissism on perceived stress through mental toughness. Personal. Individ. Differ..

[43] Erkoreka L., Navarro B. (2017). Vulnerable narcissism is associated with severity of depressive symptoms in dysthymic patients. Psychiatry Res..

[44] Jonason P.K., Webster G.D. (2010). The dirty dozen: A concise measure of the dark triad. Psychol. Assess..

[45] Czarna A.Z., Jonason P.K., Dufner M., Kossowska M. (2016). The Dirty Dozen Scale: Validation of a Polish Version and Extension of the Nomological Net. Front. Psychol..

[46] Zigmond A.S., Snaith R.P. (1983). The Hospital Anxiety and Depression Scale. Acta Psychiatr. Scand..

[47] Bjelland I., Dahl A.A., Haug T.T., Neckelmann D. (2002). The validity of the Hospital Anxiety and Depression Scale. An updated literature review. J. Psychosom. Res..

[48] Stern A.F. (2014). The Hospital Anxiety and Depression Scale. Occup. Med..

[49] Stanisz A. (2006). An Affordable Statistic Course with the Use of STATISTICA PL on the Examples of Medicine. Tom 1. Basic Statistics.

[50] Nyström M.B.T., Mikkelsen F. (2013). Psychopathy-Related Personality Traits and Shame Management Strategies in Adolescents. J. Interpers. Violence.

[51] Pailing A., Boon J., Egan V. (2014). Personality, the Dark Triad and violence. Personal. Individ. Differ..

[52] Kiejna A., Piotrowski P., Adamowski T., Moskalewicz J., Wciórka J., Stokwiszewski J., Rabczenko D., Kessler R.C. (2015). The prevalence of common mental disorders in the population of adult Poles by sex and age structure—An EZOP Poland study. Psychiatr. Pol..

[53] Zagdańska M., Kiejna A. (2016). Prevalence and risk factors of depressive episodes among student population in Wroclaw –epidemiological study results. Psychiatr. Pol..

[54] Hofmann S.G., Sawyer A.T., Fang A., Asnaani A. (2012). Dysregulation model of mood and anxiety disorders. Depress. Anxiety.

[55] Czarna A.Z., Zajenkowski M., Dufner M., Hermann A.D., Brunell A.B., Foster J.D. (2018). How Does It Feel to Be a Narcissist? Narcissism and Emotions. Handbook of Trait Narcissism: Key Advances, Research Methods, and Controversies.

[56] Zeigler-Hill V., Vonk J. (2015). Dark Personality Features and Emotion Dysregulation. J. Soc. Clin. Psychol..

[57] Birkás B., Gács B., Csathó Á. (2016). Keep calm and don’t worry: Different Dark Triad traits predict distinct coping preferences. Personal. Individ. Differ..

[58] Kong X., Kong F., Zheng K., Tang M., Chen Y., Zhou J., Li Y., Diao L., Wu S., Jiao P. (2020). Effect of Psychological–Behavioral Intervention on the Depression and Anxiety of COVID-19 Patients. Front. Psychiatry.

[59] Allroggen M., Rehmann P., Schürch E., Morf C.C., Kölch M. (2018). The Relationship Between Narcissism and Personality Traits of the Five-Factor-Model in Adolescents and Young Adults. Z. Kinder Jugendpsychiatr. Psychother..

[60] Sauls D., Zeigler-Hill V. (2020). Basic emotional systems and narcissistic personality features: What is the emotional core of narcissism?. Personal. Individ. Differ..

[61] Kroencke L., Geukes K., Utesch T., Kuper N., Back M.D. (2020). Neuroticism and emotional risk during the COVID-19 pandemic. J. Res. Personal..

[62] Nikčević A.V., Marino C., Kolubinski D.C., Leach D., Spada M.M. (2021). Modelling the contribution of the Big Five personality traits, health anxiety, and COVID-19 psychological distress to generalised anxiety and depressive symptoms during the COVID-19 pandemic. J. Affect. Disord..

